# A Comparative Study Between Fine-Needle Aspiration Cytology and Core Needle Biopsy in Diagnosing Clinically Palpable Breast Lumps

**DOI:** 10.7759/cureus.27709

**Published:** 2022-08-05

**Authors:** Kalyani Tripathi, Rita Yadav, Shyam Kumar Maurya

**Affiliations:** 1 Department of Pathology, Vivekananda Polyclinic and Institute of Medical Sciences, Lucknow, IND

**Keywords:** diagnostic accuracy, palpable lump, breast, fnac, core needle biopsy (cnb)

## Abstract

Background and objective

Most breast diseases present as palpable lumps. The accuracy of their diagnosis can be enhanced by a combination of clinical examination, mammography, fine-needle aspiration cytology (FNAC), and core needle biopsy (CNB) preoperatively. The ultimate aim of FNAC or CNB of the breast mass is to confirm the diagnosis of cancer preoperatively, which may help avoid unnecessary surgeries for benign conditions. Histopathology is the gold standard to establish the diagnosis of a breast mass. In this study, we aimed to compare the sensitivity, specificity, positive predictive value (PPV), negative predictive value (NPV), and diagnostic accuracy of FNAC with those of trucut biopsy, and provide a combined assessment of FNAC and CNB against the final histopathological diagnosis of excised breast mass in suspected breast malignancies.

Materials and methods

This was a prospective, observational, cross-sectional study conducted for a duration of one year involving 42 patients with suspected breast cancer cases who underwent FNAC, CNB, and surgical excision followed by a histopathological examination. Data were collected and analyzed in terms of sensitivity, specificity, PPV, NPV, and diagnostic accuracy of FNAC and CNB in comparison with histopathology.

Results

The majority of the patients in the study (78.6%) were in the age group of 31-50 years. In our study, FNAC showed a sensitivity, specificity, PPV, NPV, and diagnostic accuracy of 74.1%, 76.9%, 87.0%, 64.7%, and 75% respectively. CNB had a sensitivity, specificity, PPV, NPV, and diagnostic accuracy of 85.2%, 92.8%, 95.8%, 76.5%, and 87.8% respectively. The level of agreement between the two modalities (FNAC and CNB) was moderate and statistically significant (k=0.543; p<0.001). In the combined assessment of FNAC and CNB against final histopathological diagnosis for malignancy/borderline diagnosis, the sensitivity, specificity, PPV, NPV, and diagnostic accuracy were 89.3%, 85.7%, 92.6%, 80%, and 88.1% respectively.

Conclusion

The diagnostic accuracy of the combined assessment of FNAC and CNB against final histopathological diagnosis for malignancy/borderline diagnosis was better than that of FNAC or CNB alone. This finding shows that both the techniques complement each other. FNAC and CNB are used as the first- and second-line methods of pathological diagnosis respectively.

## Introduction

Breast diseases are a common cause of concern among women, and breast cancer is one of the most common causes of cancer-related deaths among women globally [1], in both the developing and developed regions [2]. The rise in the annual incidence of breast cancers of 0.5-2% has been seen across all regions of India and in all age groups, but more so in the younger age groups (<45 years) [3]. While the majority of breast cancer patients in western countries are postmenopausal and in their 60s and 70s, the picture is quite different in India with premenopausal patients constituting about 50% of all patients [4]. Decision-making in the management of breast cancer cases is dependent on the appropriate diagnosis based on the stage of the disease [5].

Preoperative diagnosis of a breast lump is a crucial part of the final therapeutic plan. The two most common techniques used to diagnose breast lumps are fine-needle aspiration cytology (FNAC) and core needle biopsy (CNB). In recent years, FNAC and CNB have been established as very efficient tools for the diagnostic evaluation of palpable breast lumps [6].

The unquestionable advantages of FNAC include its easy availability, simplicity of the technique, low cost, and most of all, low risk of complications. It requires no anesthesia, is minimally invasive, and is relatively patient-friendly [7,8]. Moreover, the results are available two to four days after aspiration. However, FNAC is associated with certain drawbacks. For instance, It can potentially mask radiological assessment when done prior to radiological investigation [9,10]. It is also possible that the smears may be acellular, making cytological analysis impossible, and these are described as inadequate aspirates. Also, its inadequacy rates vary markedly, especially since the technique is operator-dependent. These limitations of FNAC have contributed to a surge in the use of CNB [11].

The main advantage of CNB is that it provides adequate tissue for definitive histological diagnosis [12]. This method owes its growing popularity not only to its accuracy in differentiating between benign and malignant lesions but also mostly due to its capability to distinguish between an in situ lesion and invasive carcinoma [13]. Determining the type of tumors, grading tumors, and the assessment of ER and PR receptors for immunohistochemistry staining are some of the other advantages of CNB [14,15].

In view of the significance of preoperative differentiation between breast cancer types, it is essential that the common minimally invasive techniques used for this purpose should be evaluated properly in order to assess their relative clinical properties and should be traded off with respect to associated discomfort, inconvenience, and associated risks to the patient [4,5]. Hence, the present study was planned to compare the efficacy of CNB as compared to FNAC in diagnosing palpable breast lumps against the histopathological examination of the gross specimen as the gold standard.

## Materials and methods

This was a prospective, descriptive, cross-sectional study, approved by the Institutional Ethics Committee of the Vivekananda Polyclinic and Institute of Medical Sciences (VPIMS). After obtaining informed consent from the patients, the study was conducted in the Department of Pathology, VPIMS, Lucknow. A total of 42 women with clinical or radiological suspicion [Breast imaging-reporting and data system (BI-RADS III to BI-RADS V)] for malignancy were enrolled in the study and admitted to surgery and oncology wards.

The sample size was calculated by using the following formula:

n=(Zα/2)2 p (1-p)/d2 x prevalence

where, n is the required sample size, p=sensitivity, d=precision, and Z(α/2)=significance level. Assuming 80% power and 5% significance level with 0.08 precision, the calculated sample size was 42.

n=(1.96*1.96)*0.84*0.16/(0.08*0.08)*0.52=42

The inclusion criteria were as follows: all female patients with breast lumps carrying the likelihood of malignancy either clinically or radiologically. The exclusion criteria were as follows: patients with breast lumps who were not taken up for surgery, patients non-compliant for FNAC or trucut biopsy, all cases of breast lumps with no likelihood of malignancy either clinically or radiologically, and patients with bleeding disorders.

Relevant clinical history and radiological investigations were carried out among all patients. All patients underwent FNAC by the pathologist in the Department of Pathology; trucut biopsies were done by a surgeon in the minor OT and cores of the biopsy were sent to the Department of Pathology in a labeled formalin container. Both the procedures were done after obtaining informed consent from the patients.

All patients underwent surgery, and the histopathological diagnosis of the gross specimen was documented and compared with preoperative diagnostic modalities like FNAC and trucut biopsy. Smears of FNAC were stained by H&E and MGG stains and reported using the standard National Health Service Breast Screening Programme (NHSBSP) guidelines. Trucut biopsies were stained by H&E stains and reported as per standard protocol. Histopathological examination of the gross specimen was taken as the final diagnosis.

Statistical analysis was performed using Microsoft Excel 2013. Data were analyzed using SPSS Statistics 21.0 (IBM, Armonk, NY). Kappa statistic was calculated to assess the level of agreement. Sensitivity, specificity, positive predictive value (PPV), negative predictive value (NPV), and diagnostic accuracy were calculated. A p-value less than 0.05 was considered statistically significant.

## Results

The present study was carried out to compare the diagnostic efficacy of FNAC and trucut biopsy in preoperative assessment of suspected breast cancer cases. For this purpose, a total of 42 patients fulfilling the eligibility criteria were enrolled in the study. Table 1 shows the age profile of the patients enrolled in the study. The age of patients ranged from 30 to 76 years. The majority of the patients were aged 31-50 years (78.6%). All the participants in the study were females.

**Table 1:** Age profile of patients enrolled in the study SD: standard deviation

SN	Age group	No. of patients	Percentage
1	≤30 years	1	2.4
2	31-40 years	15	35.7
3	41-50 years	18	42.9
4	51-60 years	3	7.1
5	>60 years	5	11.9
Mean age of patients ±SD (range) in years		46.60 ±10.64 (30-76)	

As shown in Table 2, FNAC diagnosis of C1, C2, C3, C4, and C5 was made in two (4.8%), nine (21.4%), eight (19.0%), six (14.3%), and 17 (40.5%) cases respectively.

**Table 2:** Distribution of cases according to FNAC diagnostic category FNAC: fine-needle aspiration cytology

SN	FNAC diagnosis	No. of patients	Percentage
1	C1	2	4.8
2	C2	9	21.4
3	C3	8	19.0
4	C4	6	14.3
5	C5	17	40.5

As shown in Table 3, two cases were categorized as C1. Among the nine cases categorized as C2, the FNAC diagnosis was fibrocystic breast disease in four, granuloma/mastitis in two, and fibroadenoma, inflammatory lesion, and galactocele in one case each. Of the eight cases categorized as C3, a total of four were fibrocystic breast disease with mild atypia, two were fibroadenoma with mild atypia, one was mucocele-like lesion with mild atypia, and one was inflammatory lesion with mild atypia. Out of the six cases categorized as C4, five were suspicious for malignancy and one was positive for atypical cells. Among 17 cases categorized as C5, 11 were diagnosed as malignant and six were identified as ductal carcinoma.

**Table 3:** FNAC descriptive diagnosis FNAC: fine-needle aspiration cytology

SN	FNAC diagnosis	No. of patients	Percentage
1	C1 – inadequate	2	4.8
2	C2	9	23.8
	Fibrocystic breast disease	4	
	Granulomatous mastitis	2	
	Fibroadenoma	1	
	Inflammatory lesion	1	
	Galactocoel	1	
3	C3	8	21.4
	Fibrocystic breast disease with mild atypia	4	
	Fibroadenoma with mild atypia	2	
	Mucocele-like lesion with mild atypia	1	
	Inflammatory lesion with mild atypia	1	
4	C4	6	14.3
	Suspected for malignancy	5	
	Positive for atypical cell	1	
5	C5	17	40.5
	Positive for malignancy	11	
	Ductal carcinoma	6	

Table 4 shows that CNB categories B1, B2, B3, B4, and B5 were seen in two (4.8%), 13 (31%), four (9.5%), four (9.5%), and 20 (47.6%) of cases respectively. There was one (2.4%) case with an inadequate specimen placed in the B1 category.

**Table 4:** Distribution of cases according to core needle biopsy diagnostic category

SN	Core needle biopsy diagnosis	No. of patients	Percentage
1	B1	1	2.4
2	B2	13	31.0
3	B3	4	9.5
4	B4	4	9.5
5	B5	20	47.6

Table 5 demonstrates that the CNB B2 category included four cases of fibroadenoma, two cases each of fibrocystic breast disease, phyllodes tumor, and lymphocytic mastitis, and one case each of benign breast disease, tubercular mastitis, and granulomatous mastitis respectively. Of the four cases categorized as B3, one each was atypical ductal hyperplasia, fibroadenoma with mild atypia, focal mild atypia, and granulomatous mastitis with atypical ductal hyperplasia. Out of four cases categorized as B4, two were suspicious for malignancies, one was suggestive of malignant phyllodes, and one was ductal hyperplasia with atypical cells suggestive of malignancy. Out of 20 cases in the B5 category, a total of nine were invasive ductal carcinoma, five were non-invasive ductal carcinoma/ductal carcinoma in situ, four were lobular carcinoma, one was invasive mucinous carcinoma, and one was carcinoma with medullary features.

**Table 5:** Descriptive core needle biopsy diagnosis for different categories

SN	Core needle biopsy diagnosis	No. of patients	Percentage
1	B1 – inadequate	1	2.4
2	B2	13	31.0
	Fibroadenoma	4	
	Fibrocystic breast disease	2	
	Phyllodes tumor	2	
	Lymphocytic mastitis	2	
	Benign breast disease	1	
	Tubercular mastitis	1	
	Granulomatous mastitis	1	
3	B3	4	9.5
	Atypical ductal hyperplasia	1	
	Fibroadenoma with mild atypia	1	
	Focal mild atypia	1	
	Granulomatous mastitis + atypical ductal hyperplasia	1	
4	B4	4	9.5
	S/o malignant phyllodes (suspicious)	1	
	Suspicious malignancy	2	
	Ductal hyperplasia with atypical cells	1	
5	B5	20	47.5
	Invasive ductal carcinoma	9	
	Non-invasive ductal carcinoma/ductal carcinoma in situ	5	
	Lobular carcinoma	4	
	Invasive mucinous carcinoma	1	
	Carcinoma with medullary features	1	

As displayed in Table 6, there was an agreement between CNB and FNAC in 28/42 cases (66.7%). With respect to CNB diagnosis, the maximum agreement was seen for B3 and B5 (75%) while the minimum for B1 (0%). For B4, the agreement was 50%, and for B2, the agreement was 61.5%. The level of agreement between the two modalities was moderate and statistically significant (k=0.543; p<0.001). Agreement=28/42=66.7%; k=0.543; p<0.001.

**Table 6:** Correlation between FNAC and CNB Diagnosis FNAC: fine-needle aspiration cytology; CNB: core needle biopsy

FNAC category	CNB diagnosis				
	B1	B2	B3	B4	B5
C1	0	1	0	0	1
C2	0	8	0	1	0
C3	0	3	3	0	2
C4	0	1	1	2	2
C5	1	0	0	1	15

After final surgery, excised breast mass was subjected to histopathological examinations, and the final confirmed diagnoses were as follows: benign (33.30%), borderline (4.80%), and malignant (61.90%), as shown in Table 7.

**Table 7:** Final histopathological diagnosis of excised breast lumps

SN	Final histopathological diagnosis	No. of patients	Percentage
1	Benign	14	33.3
2	Borderline	2	4.8
3	Malignant	26	61.9

FNAC diagnosis could be made in 40 cases. Regarding the correlation of histopathological/final diagnosis with FNAC diagnosis for malignancy/borderline, a total of 20 were found true positive, three were false positive, seven were false negative, and 10 were true negative. Correspondingly, the sensitivity, specificity, PPV, NPV and diagnostic accuracy values of FNAC for malignancy/borderline diagnosis were 74.1%, 76.9%, 87.0%, 64.7%, and 75% respectively, as shown in Table 8. k=0.472 (moderate agreement).

**Table 8:** Sensitivity, specificity, PPV, NPV, and diagnostic accuracy of FNAC against histopathology PPV: positive predictive value; NPV: negative predictive value; FNAC: fine-needle aspiration cytology

Sensitivity	Specificity	PPV	NPV	Accuracy
74.1	76.9	87.0	64.7	75.0

CNB diagnosis could be made in 41 cases. In terms of the correlation of histopathological/final diagnosis with CNB diagnosis for malignancy/borderline, a total of 23 were true positive, one was false positive, four were false negative, and 13 were true negative. Correspondingly, the sensitivity, specificity, PPV, NPV, and diagnostic accuracy of trucut biopsy for malignancy/borderline diagnosis were 85.2%, 92.8%, 95.8%, 76.5%, and 87.8% respectively, as shown in Table 9. k=0.742 (substantial agreement).

**Table 9:** Sensitivity, specificity, PPV, NPV, and diagnostic accuracy of CNB against histopathology PPV: positive predictive value; NPV: negative predictive value; CNB: core needle biopsy

Sensitivity	Specificity	PPV	NPV	Accuracy
85.2	92.9	95.8	76.5	87.8

A combined assessment (FNAC + CNB) could be made in all the cases. Regarding the correlation of histopathological/final diagnosis with a combined diagnosis for malignancy/borderline, a total of 25 were true positive, three were false positive, four were false negative, and 11 were true negative. Correspondingly, the sensitivity, specificity, PPV, NPV, and accuracy values of the combined assessment for malignancy/borderline diagnosis were 89.3%, 85.7%, 92.6%, 80%, and 88.1% respectively, as shown below in Table 10. k=0.737 (substantial agreement).

**Table 10:** Sensitivity, specificity, PPV, NPV, and diagnostic accuracy of combined assessment of FNAC + CNB against histopathology PPV: positive predictive value; NPV: negative predictive value; FNAC: fine-needle aspiration cytology; CNB: core needle biopsy

Sensitivity	Specificity	PPV	NPV	Accuracy
89.3	85.7	92.6	80	88.1

## Discussion

Early diagnosis and subsequent early treatment are the most important determinants of mortality reduction in breast cancer cases [1]. Although the most common presentation of carcinoma breast is a painless progressive lump, the majority of breast lumps are benign in nature [2]. Imaging techniques are primarily suitable for screening purposes and most of the treatment is guided by cytological or tissue level changes revealed by minimally invasive techniques like FNAC and CNB that promise to be highly accurate and close to histopathological results [6]. However, it is debatable as to which of the two techniques is more accurate in clinical situations in terms of the cytological or tissue-level changes [7,8]. In the recent past, several studies have tried to resolve this debate. However, there is heterogeneity in results in different studies and hence the need for a first-hand experience of their relative efficacy in the diagnosis and characterization of breast masses arises [9].

A total of 42 women with clinical or radiological suspicion (BI-RADS III to BI-RADS V) for malignancy were enrolled in the current study. The age of the patients ranged from 30 to 76 years. The mean age was 46.60 ±10.64 years. Mitra et al. have reported the age range of patients as 15-69 years and found the majority (36.8%) of patients to be in the range of 30-40 years [11]. In a study by Jan et al., the mean age of the patients was 39.33 years [12]. The onset of breast cancer at a relatively younger age is a well-documented problem in India [3]. The findings of the present study also endorse the onset of breast cancer at a younger age as a characteristic finding among our patients.

As the inclusion criteria of the present study were based on radiological grading, there is a possibility of having some cases with a non-malignant clinical diagnosis. The usefulness of FNAC and CNB in the characterization of both benign and malignant breast disorders is well-established. A clinical diagnosis of benign disorder along with a suspicious radiological diagnosis also endorses the role of FNAC and CNB to settle the discrepancy. Both clinical and radiological diagnoses necessarily do not match in every case, and this could be the reason why almost half of our patients were clinically diagnosed as benign (45.2%).

A comparative assessment of the present study with the previous studies shows that the previous studies have shown the inadequacy rate to range from 0% to 6% for FNAC and from 0% to 7.5% for CNB [16-18]. In the study by Saha et al., there was no case of inadequate specimen in either of the two groups [13]. All the other studies, except that of Shah et al., showed CNB inadequacy to be more common than FNAC inadequacy [19]. The findings of the present study are similar to the study by Shah et al. and show the inadequacy of specimens to be more common in FNAC as compared to that in CNB.

With respect to benign and malignant diagnoses, Mitra et al., Saha et al., and Verma et al. have shown the proportion of malignancy to be higher than that of benign diagnoses for both the techniques [11,13]. Tikku and Umap, on the other hand, reported the detection of benign cases to be higher than malignant diagnoses for FNAC, whereas for CNB, like other studies, they also observed the rate to be higher for malignant as compared to that for benign diagnoses [14]. In contrast, Shah et al. found the detection of malignancy to be higher than benign diagnoses for both FNAC as well as CNB [19]. As far as the adequacy of the specimen is concerned, almost all the studies have shown a relatively much lower inadequacy rate for both the techniques and the differences in inadequacy rates for the two techniques seem to be incidental only. With respect to the difference in the proportion of benign and malignant cases, most of the studies showed a similar trend for both techniques. However, with respect to the difference in the proportion of different diagnoses in a study, it may be attributable to different patient characteristics and their clinicopathological profiles in each of these studies [17,18].

In the present study, for individual cytological and biopsy diagnoses (C1, C2, C3, C4, and C5 vs. B1, B2, B3, B4, and B5), there was an agreement between CNB and FNAC diagnosis in 28/42 cases (66.7%). The level of agreement between the two was moderately significant. Compared to the present study, a much lower agreement between CNB and FNAC was reported by Tikku and Umap who found this agreement in 58/107 (54.2%) cases only [14]. Verma et al. in their study assessed the agreement for different corresponding cytological and biopsy categories and found it to range from poor (C1) to near-perfect (C2) [20]. In another study, Siddavatan et al. found a moderate concordance between FNAC and CNB, similar to the present study [16,21]. Krishna et al. in their study reported a disagreement between FNAC and CNB diagnosis in 11/58 (19.0%) cases and found an agreement between the two in the remaining 81% of cases [17]. The heterogeneity in the level of agreement between FNAC and CNB in different studies could be attributed to individual patient characteristics as well as the method of assessment of this agreement. In the present study, we assessed this agreement between FNAC and CNB based on reporting categories whereas some authors like Krishna et al. assessed it for the compatible histopathological diagnosis [17].

In this study, the final diagnosis was prepared with the help of histopathology. Biopsy specimens obtained by CNB have a sufficient quantity of RNA/DNA on which molecular testing can be done, whereas, in the case of FNAC, this is not possible in most cases [14].

In the present study, after the surgical excision of breast lumps, the final histopathological diagnosis was benign in 14 (33.3%), borderline in two (4.8%), and malignant in 26 (61.9%) cases. Fibroadenoma was the most common benign diagnosis while invasive ductal carcinoma was the most common malignant diagnosis. The malignancy rate in various previous studies ranged from 48.4% to 84% [13,15]. The diagnosed malignancy rate in the present study was in between these two extremes. Histopathologically, all the studies diagnosed infiltrating/invasive ductal carcinoma as the most common malignant type while fibroadenoma was the most common histopathological type for benign masses. Thus, the findings of the present study are in agreement with the observations of the contemporary evidence in different studies and do not show any discrepancy.

Most of the other studies evaluated the agreement between two modalities for broad diagnosis of malignant and benign lesions and generally used histopathological outcomes as the gold standard for evaluation. In the present study, we also focused on this aspect. The sensitivity and specificity of FNAC and CNB for the detection of malignancy/borderline lesions in different studies and their comparisons with the present study are presented in Table 11.

**Table 11:** Sensitivity and specificity of FNAC and CNB reported in various contemporary studies and their comparison with the present study FNAC: fine-needle aspiration cytology; CNB: core needle biopsy

SN	Study	Sample size	FNAC			CNB/trucut	
			Sensitivity	Specificity	Sensitivity		Specificity
1	Mitra et al. [11]	68	97.4%	72.4%	97.4%		96.4%
2	Saha et al. [13]	50	69.0%	100%	83.3%		100%
3	Tikku and Umap [14]	107	64.6%	100%	95.8%		100%
4	Garg and Yadav [16]	50	92%	96%	96%		100%
5	Krishna et al. [17]	54	86.4%	93.8%	95.5%		100%
6	Shah et al. [19]	50	70.8%	96.2%	87.5%		100%
7	Present study	42	74.1%	76.9%	85.2%		92.9%

As for the sensitivity of FNAC, it has been found to range from 64.6% to 97.4% in different studies, while its specificity has been reported to range from 72.4% to 100% [11,13,14]. However, in the present study, we had both sensitivity and specificity close to 75%, which otherwise has not been assessed in the previous studies. With respect to the sensitivity of CNB, it has been reported to range from 83.3% to 97.4% while specificity has been reported to range from 94.2% to 100% [11,13,14,16-18]. Compared to these studies, in the present study, both sensitivity and specificity were of lower order.

Thus, in the present study, for both FNAC as well as CNB, the diagnostic accuracy was lower as compared to the prior studies [11-17]. The reason for this could be the small sample size and difference in malignancy rates. In the present study, the malignancy rate was 66.7%, which is second only to that reported by Saha et al. [13]. Owing to this high malignancy rate, the percentage value of each misdiagnosis was high and a chance of misdiagnosis in cases of benign types resulted in a decline in specificity by 7.1%. Moreover, the high sensitivity in some studies might be due to the inclusion of doubtful (B3/C3) diagnoses as positive observations. A change in criteria for the determination of malignancy might have a significant impact on diagnostic performance.

Despite these differences in terms of diagnostic efficacy compared to previous studies, the present study aligns with other studies in that it found CNB to have both higher sensitivity as well as specificity as compared to FNAC. Thus, despite the fact that both the techniques had a lower diagnostic efficacy as compared to the previous studies due to the reasons cited above, the findings of the present study are in agreement with the previous studies that have shown CNB to have a slight edge over FNAC.

In the present study, we also carried out a combined assessment by considering positive diagnosis by either FNAC or CNB as malignant, which helped in two ways: firstly, it was helpful in making a diagnosis in all the cases (two cases missed on FNAC and one case on CNB could be diagnosed with the help of alternative diagnosis). Secondly, it was also helpful in increasing the sensitivity to 89.3%, which is higher than the sensitivity of either of the two individual diagnoses; moreover, we achieved a specificity of 85.7%, which is higher than that of FNAC (74.1%) but lower than that of CNB (92.9%). In effect, it was helpful in diagnosing at least two more cases of malignancy than either of the two techniques could independently do. Given that FNAC and biopsy provide different information regarding the cytological and tissue architectural changes, they seem to have a complementary rather than a competitive role, and using them together could help in achieving a better diagnostic yield.

## Conclusions

Based on our findings, CNB is slightly better than FNAC for the diagnosis of malignant/borderline breast masses. The combined use of both modalities was helpful in dealing with the problem of inadequacy of specimens and also provided more sensitive and accurate outcomes as compared to diagnosis by either of the two methods alone. The findings of the present study endorse the viewpoint that both the techniques are complementary and the combined use of both techniques might help in obtaining higher diagnostic accuracy.
